# Molecular basis of anti-CRISPR operon repression by Aca10

**DOI:** 10.1093/nar/gkac656

**Published:** 2022-08-03

**Authors:** So Yeon Lee, Nils Birkholz, Peter C Fineran, Hyun Ho Park

**Affiliations:** College of Pharmacy, Chung-Ang University, Seoul 06974, Republic of Korea; Department of Global Innovative Drugs, Graduate School of Chung-Ang University, Seoul 06974, Republic of Korea; Department of Microbiology and Immunology, University of Otago, PO Box 56, Dunedin 9054, New Zealand; Bioprotection Aotearoa, University of Otago, PO Box 56, Dunedin 9054, New Zealand; Department of Microbiology and Immunology, University of Otago, PO Box 56, Dunedin 9054, New Zealand; Bioprotection Aotearoa, University of Otago, PO Box 56, Dunedin 9054, New Zealand; College of Pharmacy, Chung-Ang University, Seoul 06974, Republic of Korea; Department of Global Innovative Drugs, Graduate School of Chung-Ang University, Seoul 06974, Republic of Korea

## Abstract

CRISPR-Cas systems are bacterial defense systems for fighting against invaders such as bacteriophages and mobile genetic elements. To escape destruction by these bacterial immune systems, phages have co-evolved multiple anti-CRISPR (Acr) proteins, which inhibit CRISPR-Cas function. Many *acr* genes form an operon with genes encoding transcriptional regulators, called anti-CRISPR-associated (Aca) proteins. Aca10 is the most recently discovered Aca family that is encoded within an operon containing *acrIC7* and *acrIC6* in *Pseudomonas citronellolis*. Here, we report the high-resolution crystal structure of an Aca10 protein to unveil the molecular basis of transcriptional repressor role of Aca10 in the *acrIC7-acrIC6-aca10* operon. We identified that Aca10 forms a dimer in solution, which is critical for binding specific DNA. We also showed that Aca10 directly recognizes a 21 bp palindromic sequence in the promoter of the *acr* operon. Finally, we revealed that R44 of Aca10 is a critical residue involved in the DNA binding, which likely results in a high degree of DNA bending.

## INTRODUCTION

As a result of long evolutionary battle, bacteria and archaea have evolved clustered regularly interspaced short palindromic repeats (CRISPR) and CRISPR-associated protein (Cas) (CRISPR-Cas) systems for fighting against invaders such as phages (bacterial viruses) and other mobile genetic elements (MGEs) (1–3). CRISPR-Cas are adaptive immune systems because they record information of the genetic material of invaders, which is then used to detect and destroy these invaders during subsequent infections (1,4,5). CRISPR-Cas systems have diversified into two classes and six types (I–VI) (6). The most prominent difference between the two classes is whether the Cas proteins form multi-subunit protein complex (class 1), or whether a single multi-domain Cas protein contains all required activities (class 2) (7,8). Types I, III, and IV belong to class 1, while types II, V and VI belong to class 2.

To escape from the CRISPR-Cas adaptive immune system, phages and MGEs have evolved multiple strategies (9). One common strategy is provided by anti-CRISPR (Acr) proteins that can neutralize the host CRISPR-Cas defense system through various mechanisms (10–14). The first Acrs were identified in phages as small proteins that inhibited the activity of the type I-F CRISPR-Cas system of *Pseudomonas aeruginosa* (15). Since then, more than 60 Acr proteins have been discovered by functional screening and bioinformatic analyses (10,16). Due to extensive diversity of Acr proteins and a lack of common structural motifs, Acrs are classified based on the CRISPR-Cas system targeted (8,10). For example, if Acr proteins inhibit the type I-F CRISPR-Cas system, they are named AcrIF (e.g. AcrIF4), and if Acr proteins block the activity of type II-A CRISPR-Cas, they are denoted AcrIIA (17).

Although the sequence and genomic locations of *acr* genes are diverse, many *acr* genes form an operon with conserved genes encoding putative transcriptional regulators with DNA binding domain, called anti-CRISPR-associated (Aca) proteins (18,19). So far, ten Aca families have been identified in various phages and archaeal viruses (15,20–25). For example, *Pseudomonas aeruginosa* phage JBD30 contains an *acrIF1–aca1* operon and *Pectobacterium carotovorum* phage ZF40 contains an *acrIF8–aca2* operon (18,19). Although the exact function of Aca proteins is still under investigation, recent studies indicated that they can serve as transcriptional repressors to inhibit the production of Acr proteins (18,19). The working mechanisms of Aca1 and Aca2 were revealed by recent genomic and structural studies (18,19,26–28). Both Aca1 and Aca2 block transcription of *acr* genes by direct interaction with specific inverted repeats (IRs) around the promoter of *acr* genes, indicating that transcription inhibition by Acas occurs by blocking the recruitment of RNA polymerase to the promoter. Interestingly, in some cases, including AcrIIA1, AcrIIA13, AcrIIA15 and AcrIF24, Acrs themselves can possess Aca activity by containing a DNA binding domain for autoregulation (23,29,30). It has also been reported that bacteria may contain their own Aca-like proteins to inhibit Acr expression by invaders and to maintain CRISPR-Cas defense systems (19,29).

Aca10 is the most recently identified Aca family that is suggested as a transcriptional regulator by controlling the expression of *acrIC7* and *acrIC6* in *P. citronellolis* and in other analogous contexts (20). To elucidate the molecular basis of transcriptional control of *acr* genes by Aca10, in this study, we determined the high-resolution crystal structure of Aca10 from *P. citronellolis* (pcAca10) and revealed the molecular mechanism underlying promoter recognition by Aca10 for regulating the expression of *acr* genes. This study increases our understanding of Aca-mediated control of anti-CRISPR expression for CRISPR-Cas inactivation during bacterial takeover by phages.

## MATERIALS AND METHODS

### Protein expression and purification

The *aca10* gene from *Pseudomonas citronellolis* (accession code: TGC30851.1) was synthesized by Bionics (Daejeon, Republic of Korea) and then cloned into a pET21a vector (Novagen, Wisconsin, USA), which contains a C-terminal hexa-histidine tag for affinity chromatography. The NdeI and XhoI restriction sites were utilized for cloning. The resulting recombinant vector encoding full-length Aca10 (residue 1–65) was transformed into competent *Escherichia coli* BL21(DE3) cells for transformation. A single colony was selected and cultured in 5 ml lysogeny broth (LB) medium containing 50 μg/ml ampicillin overnight at 37°C, after which 1 ml of the culture was transferred to 1 l of LB for large culture. When the optical density measured at 600 nm reached 0.7–0.8, the temperature was adjusted to 20°C and 0.25 mM isopropyl β-d-1-thiogalactopyranoside (IPTG) was added for *aca10* expression. The induced cells were further cultured for 18 h at 20°C in a shaking incubator. Cultured cells were harvested by centrifugation at 3500 g for 15 min at 20°C. Then, the pelleted cells were resuspended in lysis buffer (20 mM Tris–HCl (pH 8.0) and 500 mM NaCl) and lysed via ultrasonication at 4°C. The cell lysate and the supernatant were separated by centrifugation at 14 000 g for 30 min at 4°C. The supernatant was collected and mixed with Ni-nitrilotriacetic acid (NTA) affinity resins and incubated for 2 h at 4°C. The incubated mixture was loaded onto a gravity-flow column (Bio-Rad, Hercules, CA, USA). The resin in the gravity column was washed with 50 ml lysis buffer to remove impurities. After washing, the resin-bound Aca10 protein was eluted using elution buffer (20 mM Tris–HCl (pH 8.0), 500 mM NaCl, 250 mM Imidazole). The Aca10 sample was purified by size-exclusion chromatography (SEC) on a Superdex 200 Increase 10/300 GL column (GE Healthcare, Waukesha, WI, USA) pre-equilibrated with SEC buffer (20 mM Tris–HCl (pH 8.0) and 150 mM NaCl). The target protein Aca10 eluted from SEC was collected, pooled, and concentrated to 7.4 mg/ml for crystallization. Purity of the protein was visually assessed using sodium dodecyl sulfate-polyacrylamide gel electrophoresis (SDS-PAGE).

### Crystallization and data collection

Aca10 was crystallized using the hanging drop vapor diffusion method at 20°C. Initial crystals were obtained by equilibrating a mixture containing 1 μl of protein solution (7.4 mg/ml protein in SEC buffer) and 1 μl of a reservoir solution containing 10% (v/v) 2-propanol, 0.1 M Imidazole pH 8.0, against 0.3 ml of reservoir solution. We obtained crystals in 24 h. A single crystal was selected and soaked in the reservoir solution supplemented with 30% (v/v) glycerol for cryo-protection, X-ray diffraction data were collected at −178°C on the beamline BL-5C at Pohang Accelerator Laboratory (Pohang, Korea). Data processing including indexing, integration, and scaling, was conducted using HKL2000.

### Structure determination and analysis

The Aca10 structure was determined using the molecular replacement phasing method, which was performed using PHASER in the PHENIX package (31). The predicted structural model generated by AlphaFold2 was used as a search model (32). The initial model was built automatically using AutoBuild from the Phenix package, and further model building with refinement was performed using Coot (33) and phenix.refine (34). The structure quality and stereochemistry were validated using MolProbity (35). All structural figures were generated using PyMOL (36).

### SEC-multi-angle light scattering (MALS) analysis

The absolute molecular mass of Aca10 in solution was measured using SEC-coupled multi-angle light scattering (SEC-MALS). Purified Aca10 protein solution was loaded onto a Superdex 200 Increase 10/300 GL 24 ml column pre-equilibrated with SEC buffer. The flow rate of the buffer was maintained at 0.5 ml/min and SEC-MALS was performed at 20°C. A DAWN-Treos MALS detector (Wyatt Technology, Santa Barbara, USA) was interconnected with the ÄKTA explorer system (GE Healthcare). The molecular mass of bovine serum albumin was used as the reference value. The absolute molecular mass was assessed using the ASTRA program (Wyatt Technology).

### Mutagenesis

Site-directed mutagenesis was performed using the Quick-Change kit (Stratagene), and the resulting mutants were confirmed through sequencing. All mutant proteins were expressed and purified in the same manner as the wild type described above.

### Identification of the potential Aca10 binding site

The 5′ regulatory region upstream of the *acrIC7-acrIC6-aca10* operon from *P. citronellolis* was analyzed to identify a potential promoter using BPROM (http://www.softberry.com) and manual curation. The presence of potential Aca10 binding sites was determined using the Repeat Finder plugin in Geneious Prime version 2022.1.1 (https://www.geneious.com), followed by manual curation.

### Electrophoretic mobility shift assay with polyacrylamide gel

DNA oligonucleotides containing the inverted repeat sequences identified were synthesized by Bionics. To generate double-stranded DNA for EMSA assays, we incubated the top strand DNA oligo (100 μM) and bottom strand DNA oligo (100 μM) at 100°C for 3 min and slowly cooled to room temperature to anneal.

Varying concentrations of purified wildtype or mutant Aca10 were pre-incubated with 20 ng of double-stranded DNA in SEC buffer for 60 min on ice. Prepared samples were then separated by gel electrophoresis at 100 V on a 10% native 0.5× TBE (Tris-borate EDTA) polyacrylamide gel for 1 h. Afterwards, gels were stained with SYBR Gold (Invitrogen, Waltham, MA, USA) and visualized according to the manufacturer's instructions.

### Sequence alignment

Amino acid sequences of Aca10 homologs from various species were analyzed using Clustal Omega (37).

### Modeling of Aca10/DNA complex structure

The Aca10/DNA complex was modeled using the HDOCK server (38). For predicting the complex structure of dimeric Aca10/linear DNA, the IR2 sequence, 5′-AATACGCTCATTGAGCGTATT-3′, was used as the input DNA sequence. To construct the complex between dimeric Aca10 and bent DNA, the structure of bent DNA was obtained from a previously solved structure of MqsA (PDBid: 3O9X) (39) and used for this docking study. The default docking parameters provided by HDOCK server were used.

## RESULTS AND DISCUSSION

### Structure of Aca10 from *Pseudomonas citronellolis*

**Figure 1.**
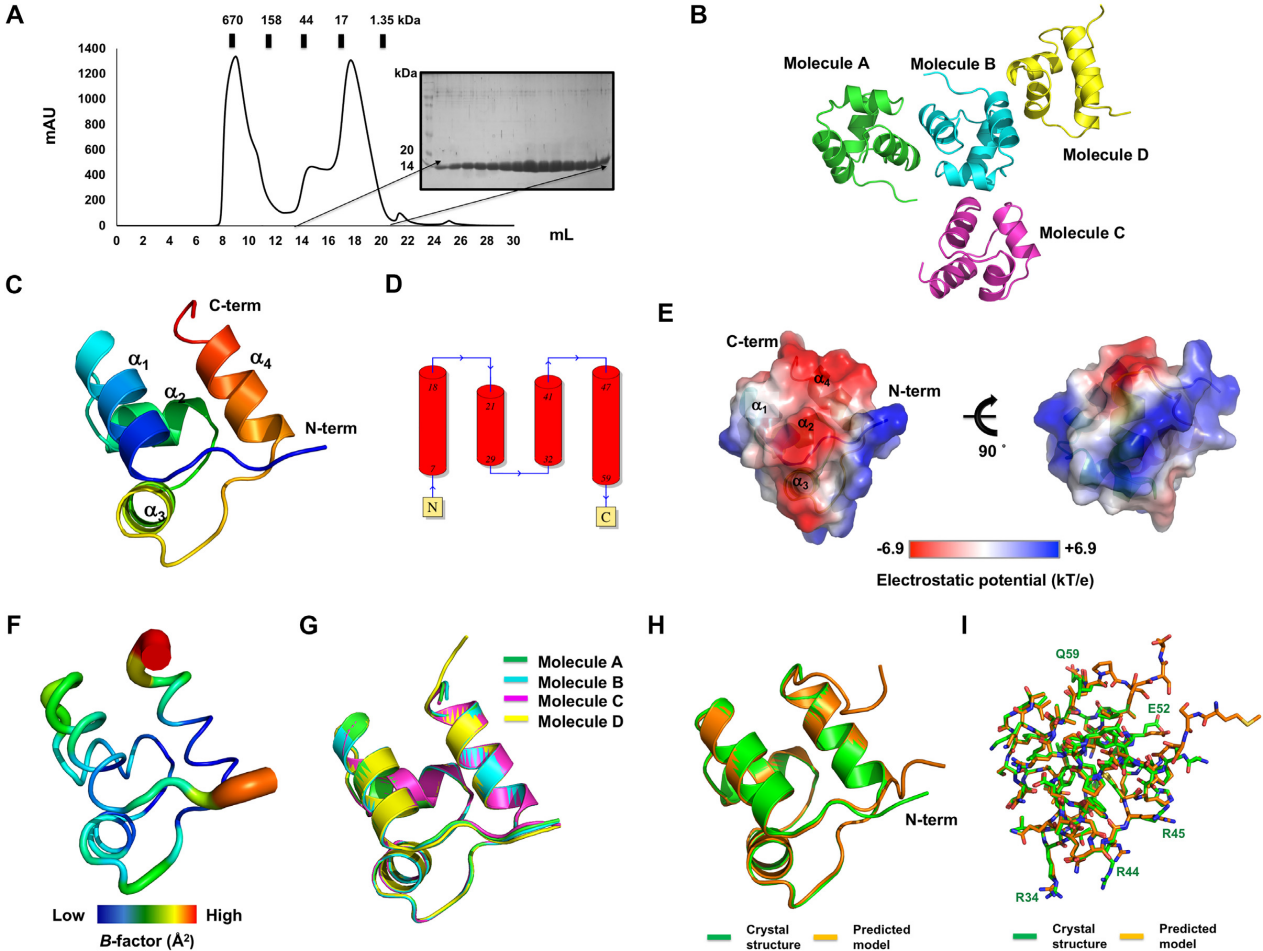
Crystal structure of Aca10 derived from *Pseudomonas citronellolis*. (**A**) Size-exclusion chromatography profile of Aca10. SDS-PAGE gel showing the protein fractions eluted at the peak position. (**B**) Cartoon representation of the structure of four Aca10 molecules presented in a crystallographic asymmetric unit. (**C**) Cartoon representation of the monomeric Aca10 structure. The color of the chain from the N- to the C-termini gradually moves through the spectrum from blue to red. (**D**) Topological representation of secondary structure of Aca10. (**E**) Surface electrostatic potential of Aca10. The scale bar ranges from − 6.9 kT/e (red) to 6.9 kT/e (blue). (**F**) *B*-factor distribution in the structure of Aca10. The structure is presented in a putty representation and rainbow colors from red to violet demonstrate the order of the *B*-factor values. (**G**) Superimposition of four molecules detected in one asymmetric unit; the colors correspond to the monomers within the asymmetric unit as shown in (B). (**H** and **I**) Structural comparison of crystal structure (green) with predicted structure (orange) generated by AlphaFold2 in cartoon (H) and stick figure (I). Those residues that were not perfectly aligned were labelled.

To understand anti-CRISPR regulation, the full-length Aca10 protein was purified using a quick two-step chromatography method, comprising Ni-NTA affinity chromatography followed by size-exclusion chromatography (SEC) for structural analysis. Although an aggregation peak was detected around the column void volume, the main peak produced at approximately 17–18 ml elution volume contained highly pure Aca10 protein when assessed by SDS-PAGE (Figure 1A). The pure target protein was successfully crystallized. The diffraction data were collected at a resolution of 1.76 Å at PAL synchrotron. Due to the absence of a structural homolog of Aca10 required for the molecular replacement (MR) phasing method, structure determination was first attempted unsuccessfully with *ab initio* phasing using ARCIMBOLDO_BORGES software (40). However, the phasing issue was resolved by MR by using an accurately generated structural model predicted by alphafold2, a recent tool that has advanced structural predictions based on machine learning (32). The final structural model of Aca10 was refined to *R*_work_ = 21.1% and *R*_free_ = 23.7%. The diffraction data and refinement statistics are summarized in Table 1. The crystal belonged to space group *P4_1_2_1_2* and four molecules were present in the asymmetric unit (ASU) (Figure 1B). The final structural model contained most of the Aca10 sequence (residues from S3 to L60 out of 65 amino acids) for all four molecules. The first two amino acids at the N-terminus and the last five amino acids at the C-terminus were not included in the final models owing to unclear electron densities (Figure 1B).

**Table 1.** Data collection and refinement statistics

**Data collection**	
Space group	*P 4_1_ 2_1_ 2*
Unit cell parameters	
*a*, *b*, *c* (Å)	*a* = 109.92, *b* = 109.92, *c* = 60.74
α, β, γ (°)	α = 90, β = 90, γ = 90
Resolution range (Å)^1^	28.29–1.76
Total reflections	987 716
Unique reflections	37 421
Multiplicity	26.4 (26.6)
Completeness (%)^a^	99.94 (99.92)
Mean *I*/σ(*I*)^a^	25.08 (1.30)
*R*_merge_ (%)^a^^,^^b^	7.95 (29.54)
Wilson *B*-factor (Å^2^)	36.24
**Refinement**	
Resolution range (Å)	28.29–1.76
Reflections	37 419
*R*_work_ (%)	20.77
*R*_free_ (%)	22.63
No. of molecules in the asymmetric unit	4
No. of non-hydrogen atoms	1984
Macromolecules	1762
Solvent	222
Average *B*-factor values (Å^2^)	38.48
Macromolecules	37.45
Solvent	46.67
Ramachandran plot:	
favored/allowed/outliers (%)	99.55/0.45/0.00
Rotamer outliers (%)	0.00
Clashscore	1.43
RMSD bonds (Å)/angles (°)	0.008/0.96

^a^Values for the outermost resolution shell are shown in parentheses.

^b^ *R*_merge_ = Σ_h_ Σ_i_ |*I*(*h*)_i_ − <*I*(*h*)>|/ Σ*_h_* Σ*_i_* I(*h*)*_i_*, where *I*(*h*) is the observed intensity of reflection *h*, and < *I*(*h*)> is the average intensity obtained from multiple measurements.

The structure of Aca10 showed that it was constructed of an α-helical bundle fold comprising four α-helices (α_1_−α_4_) (Figure 1C and D). Analysis of the surface electrostatic potential of Aca10 showed that most was positively charged, whereas one face composed of α_4_ and a connected loop was highly negatively charged (Figure 1E). *B*-factor analysis showed that most of the structure was rigid with a low *B*-factor (average 36.58 Å^2^), except for both terminal regions (average 54.32 Å^2^) (Figure 1F). The structure of four molecules in one ASU was almost identical, with a root-mean-square-deviation (RMSD) value of 0.24–0.48 Å among each other (Figure 1G).

Because we solved the structure of Aca10 based on the predicted structural model generated by alphafold2, we were curious how similar between these structures were. To answer this, we compared the two structures with structural superposition. This structural comparison indicated that the main backbone of the predicted structure was nearly identical with that of the crystal structure except the N-terminal region (Figure 1H). In the case of the side chains of each residues of predicted model, several charged residues exposed on the surface were not identical with those of crystal structure (Figure 1I), indicating that the flexible side chains exposed to the surface were not accurately predicted. However, the overall backbone structure predicted by alphafold2 was almost identical with the crystal structure. In summary, Aca10 is a small four helical bundle protein with a highly charged surface.

### Aca10 forms a dimer in solution

**Figure 2.**
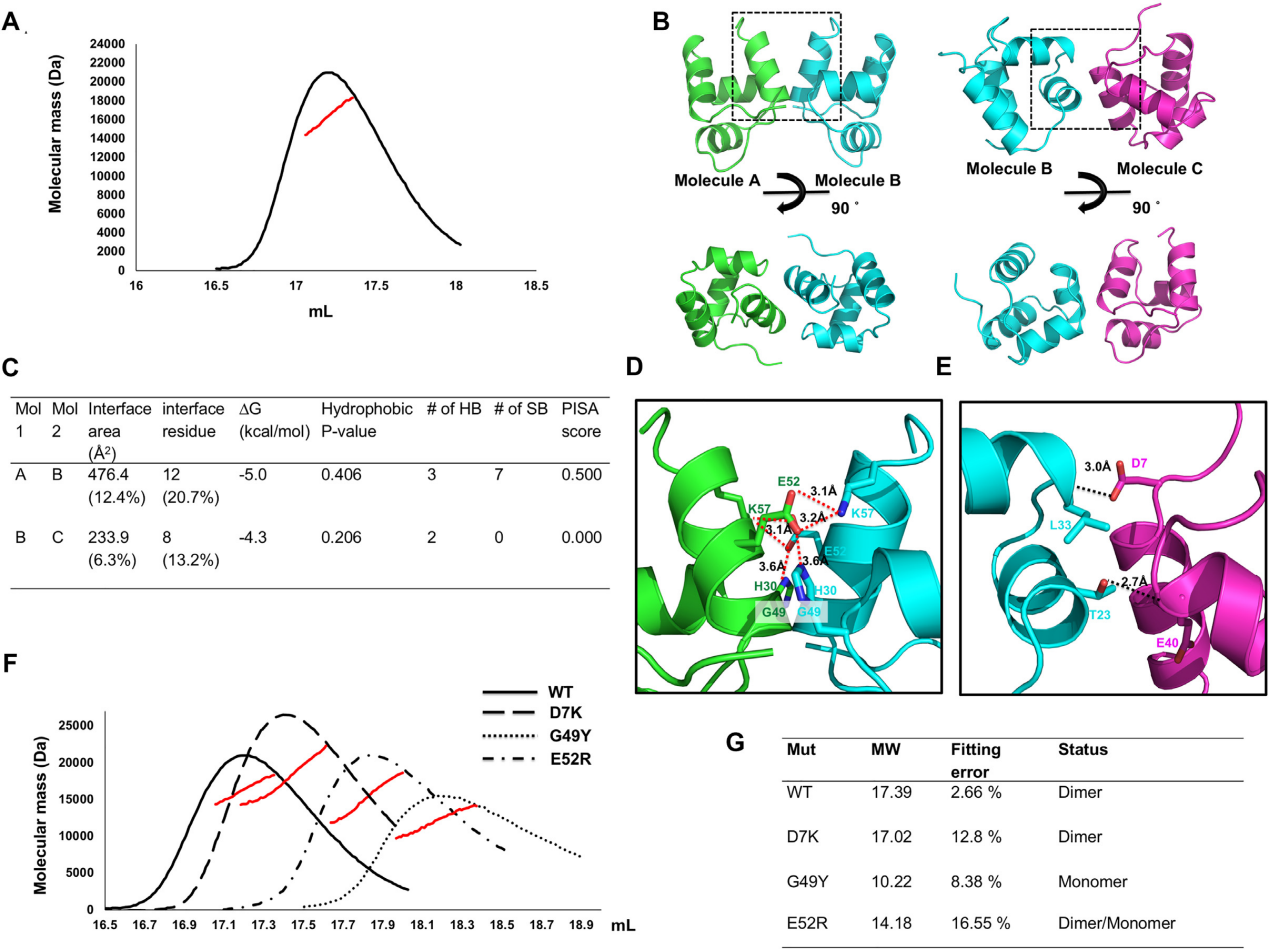
Dimeric structure of Aca10 and analysis of its interface and conformation. (**A**) Multi-angle light scattering (MALS) profile of Aca10 corresponding to the main peak of the SEC. The red line indicates the experimental molecular weight analyzed by MALS. (**B**) Crystallographic packing symmetry analysis. Two possible types of Aca10 dimer (AB and BC dimers) found in the crystallographic asymmetric unit are shown. PPI regions are indicated by black dotted boxes. (**C**) Table summarizing the PPI details of the two types of dimers analyzed by the PISA server. (**D** and **E**) Magnified view of the PPI highlighted in panel (B) for the AB dimer (D) and the BC dimer (E). Salt bridges and hydrogen bonds are indicated by red dotted lines and black dotted lines, respectively. (**F**) Verification of the PPI via mutagenesis. SEC-MALS profiles comparing the position of eluted peaks between wildtype and mutant proteins. The red line indicates the experimental molecular mass analyzed by MALS. (**G**) Table summarizing the result of MALS and mono- or dimeric status of each mutant. MW indicates molecular weight and fitting error indicate the MALS fitting error.

Initial genetic and biochemical studies of Aca family members suggested a dimeric form (18,19), with recent structural studies of Aca1 and Aca2 confirming that both Acas function as dimers in solution (26–28). However, it is not known if all Acas function as dimers and the stoichiometry of Aca10 is unknown. Since Aca stoichiometry likely influences their mechanism, we determined the exact stoichiometry of Aca10 in solution by calculating the absolute molecular mass using multi-angle light scattering (MALS). Although several peaks were detected in the SEC experiment, the molecular mass of the main peak was calculated as 17.39 kDa (2.66% fitting error) by MALS (Figure 2A). Considering that the theoretically calculated molecular weight of Aca10 was 8.2 kDa, the experimental molecular mass analyzed by MALS demonstrated that Aca10 formed a dimer in solution.

Because we observed Aca10 dimers in solution, we analyzed the contact features of the four molecules found in the crystallographic asymmetric unit to further understand dimerization. Two putative dimeric structures may form between molecule A and molecule B (AB dimer) or between molecule B and molecule C (BC dimer) (Figure 2B). The AB dimer was a symmetric dimer, whereas the BC dimer was asymmetric. Since DNA-binding proteins, especially those recognizing palindromic DNA sequences, are typically symmetric dimers, the AB dimer was the more likely biologically relevant form. To understand dimer formation of Aca10 in detail, we analyzed the protein-protein interactions (PPI) in both the AB dimer and the BC dimer using the PDBePISA PPI-calculating server (Figure 2C). In the AB dimer, the total dimer surface buried an area of 476.4 Å^2^, which represents 12.4% of the total surface area. A total of 12 residues, which represents 20.7% of the total Aca10 residues, were involved in the formation of PPI (Figure 2C). The PPI, mainly constructed by helix α_4_ from both molecules, was formed by multiple hydrogen bonds and salt bridges (Figure 2D). Salt bridges formed by residues H30, E52 and K57 from each molecule were the primary forces maintaining the integrity of this dimeric interface. Residues G49 from each monomer were the most closely localized residues between the two molecules. In the case of the BC dimer, the total dimer surface buried was 233.9 Å^2^, which represents 6.3% of the total surface area calculated by PDBePISA (Figure 2C). Only two hydrogen bonds, formed between L33 and T23 from one molecule and D7 and E40 from another molecule, were formed in the BC dimer (Figure 2E). The PISA score, which ranges from 0 to 1 as the relevance of the interface to complex formation increases, was 0.500 for the AB dimer but 0.000 for the BC dimer, further indicating that the AB dimer was the more likely biologically relevant form.

To experimentally determine which dimer was relevant, we mutagenized Aca10 and examined dimer disruption. To test the AB dimer, a G49Y and E52R mutant were generated. Residue D7, which was predicted to be involved in the BC dimer, was also mutated to test a possible effect on dimer formation. These three mutants and wild-type Aca10 were subjected to SEC to analyze dimer formation. As expected, the D7K variant (BC dimer disruption mutant) did not affect dimer formation whereas the G49Y mutant disrupted dimer formation as evidenced by delayed elution from SEC corresponding to the size of a monomer (Figure 2F and G). The absolute molecular weight of the G49Y mutant was further examined by MALS. The calculated weight of 10.22 kDa (8.38% fitting error), confirmed that the G49Y mutation results in an Aca10 monomer and demonstrated the importance of the AB dimer interface. In the case of the E52R mutant, it produced a main SEC peak around 17.8 mL in between the dimeric wild-type peak and the monomeric G49Y mutant peak. This might be due to a weak disruption effect of the E52R mutation. Although residue E52 was the main residue that mediated the PPI formation of the AB dimer, mutagenesis to arginine was not as effective as the G49Y mutant. Because of the weak disruption effect, the E52R peak might contain a mix of both monomer and dimer, which resulted in the apparent sizes. All the experimental results from SEC-MALS are summarized in Figure 2G. Based on the structural analysis, examination of crystal packing, and mutagenesis studies followed by the dimer disruption assay, we concluded that Aca10 exists as a symmetric AB dimer in solution.

### Aca10 directly binds to the promoter of the putative *acrIC6* and *acrIC7* genes

**Figure 3.**
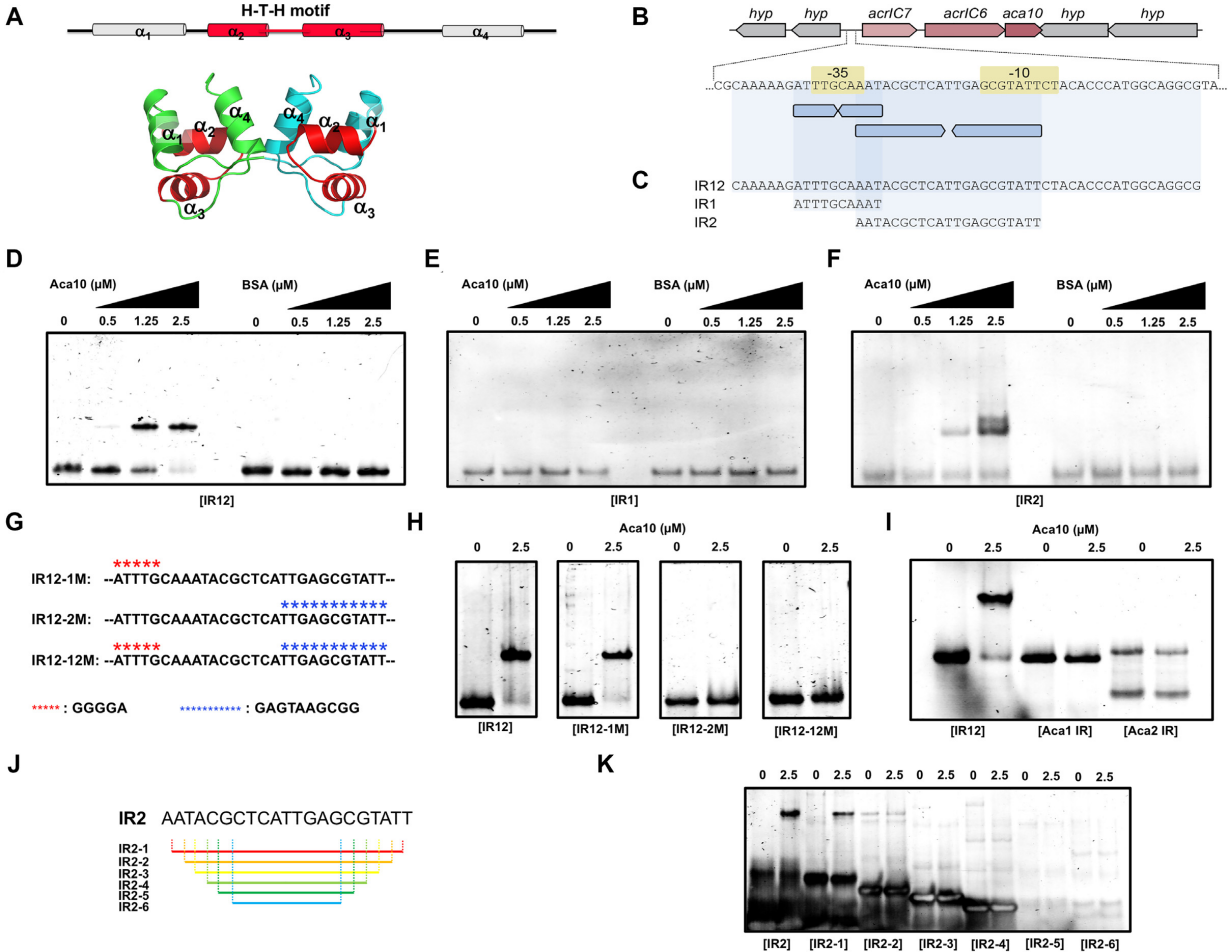
Promoter identification and specific interaction by Aca10. (**A**) The location of the tentative H-T-H motif on the structure of Aca10. (**B**) The *acrIC7-acrIC6-aca10* operon in its genomic context, with the predicted regulatory region, including -35 and -10 promoter elements, highlighted underneath. Two pairs of inverted repeats (IRs) are indicated by blue arrows. (**C**) Sequences of the IR12, IR1 and IR2 DNA probes used in subsequent experiments. (**D-F**) EMSAs of the IR12 DNA probe (D), IR1 DNA probe (E), and IR2 DNA probe (F) with increasing concentrations of Aca10 indicated by black triangles. Bovine serum albumin (BSA), which is not expected to bind DNA, was used as a negative control. (**G**) Mutated sites on the IR12 DNA fragment. Mutated sequence for IR1 disruption and IR2 disruption are indicated by red stars and blue stars, respectively. (**H**) EMSAs of the mutated IR12 DNA probes with no or 2.5 μM Aca10. (**I**) EMSAs of other inverted DNA probes with no or 2.5 μM Aca10. Aca1 IR: ACAAGCGGCACACTGTGCCTATTGCGAATTAGGCACAATGTGCCTAATCTAACG and Aca2 IR: CACTGTTCGCAATTGCGAACTAAGATGGAACCAGATTCGAGATTGGCTCGAATCACCTC. (**J**) A series of truncated IR2 oligoes tested for binding to Aca10. (**K**) EMSA of a series of truncated IR2 oligos with no or 2.5 μM Aca10

Previously studied Acas, Aca1 from *P. aeruginosa* phage JBD30 and Aca2 from *Pectobacterium carotovorum* phage ZF40, form dimers and directly interact with inverted repeats (IRs) within promoter regions of their *acr* operons to repress expression (18,19). Aca10 is predicted to be a helix-turn-helix (H-T-H) transcription regulator controlling the production of AcrIC7 and AcrIC6 in *P. citronellolis* (20) (Figure 3A). Based on this prediction and the dimeric nature of Aca10, we hypothesized that Aca10 would bind inverted repeat regions within the promoter to control transcription. To elucidate the molecular mechanism underlying promoter recognition by Aca10 for regulating the expression of *acr* genes, we first examined the region 5′ of the *acrIC7-acrIC6-aca10* operon for a predicted promoter using BPROM. This revealed −35 and −10 regions consistent with a strong promoter (TTGCAA-N16-TATTCT) (Figure 3B). Overlapping the −35 region was a short 10 bp IR consistent of two perfectly matching 5 bp half sites (IR1; ATTTG CAAAT) (Figure 3B and C). In addition, positioned within the promoter, and overlapping the −35 and −10 elements, was a 21 bp IR consisting of two perfect 10 bp half-sites (underlined) separated by 1 bp (IR2; AATACGCTCA T TGAGCGTATT) (Figure 3B and C). The position of IR1 and IR2 strongly suggests that Aca10 binding would occlude RNAP binding and lead to transcriptional repression.

Direct interaction of Aca10 and the promoter was analyzed by EMSAs using DNAs containing the different IRs. Purified Aca10 displayed concentration-dependent binding to a DNA fragment that contained both the IR1 and IR2 sequences (IR12, Figure 3C), but this was not bound by a BSA negative control (Figure 3D). Therefore, Aca10 directly binds DNA containing this sequence in *acrIC7-acrIC6-aca10* promoter of *P. citronellolis*. Next, we investigated the individual contributions of IR1 and IR2. No Aca10-dependent shift of the IR1 DNA was detected, demonstrating that IR1 was not sufficient for Aca10 binding (Figure 3E). In contrast, when the IR2 DNA fragment was incubated with Aca10, it produced a similar concentration-dependent shift as the entire IR12 fragment, indicating that IR2 is the relevant Aca10 binding site (Figure 3F). In summary, Aca10 is a dimeric H-T-H protein that binds to a 21-bp IR overlapping the *acrIC7-acrIC6-aca10* promoter.

Next, we tested the sequence-specificity and requirements of IR1 and IR2 for Aca10 binding to the *acrIC7-acrIC6-aca10* operon. For this experiment, three mutant DNA fragments, IR12-1M (IR1 disruption), IR12-2M (IR2 disruption), and IR12-12M (both IR1 and IR2 disruption), were generated (Figure 3G). Aca10 EMSAs were performed with these three mutated DNA fragments, whose inverted repeat sequences were destroyed and did not contain any inverted sequences. Although IR12 and IR12-1M produced shifts when incubated with Aca10, neither IR12-2M and IR12-12M produced shift with Aca10, indicating that Aca10 recognized the specific inverted IR2 sequence but not IR1 (Figure 3H).

We also wondered if Aca10 can only recognize the specific inverted repeat sequence identified in this study or also other recognize inverted repeat sequences of similar size. To test this specificity, we performed EMSAs with two more reverted repeat DNA oligos, Aca1 binding IR (ACAAGCGGCACACTGTGCCTATTGCGAATTAGGCACAATGTGCCTAATCTAACG) and Aca2 binding IR (CACTGTTCGCAATTGCGAACTAAGATGGAACCAGATTCGAGATTGGCTCGAATCACCTC) identified in recent studies (18,26,28). This experiment clearly showed that Aca10 failed to bind Aca1 IR and Aca2 IR (Figure3I), indicating that Aca10 specifically recognizes the IR sequence in the promoter of *acrIC7-acrIC6-aca10* operon.

Finally, we determined the minimal IR2 sequence that can be recognized and bound by the Aca10 dimer. To analyze this, we generated a series of truncated IR2 oligos (Figure 3J) and performed EMSAs with Aca10 (Figure 3K). This experiment showed that truncated IR2 oligos, with one base at both ends removed, was able to still bind to Aca10 similar to the IR2 oligo. Therefore, the minimal DNA sequence required for Aca10 binding is 5′ATACGCTCATTGAGCGTAT3′ (Figure 3K).

### Aca10 dimerization is critical for promoter recognition

**Figure 4.**
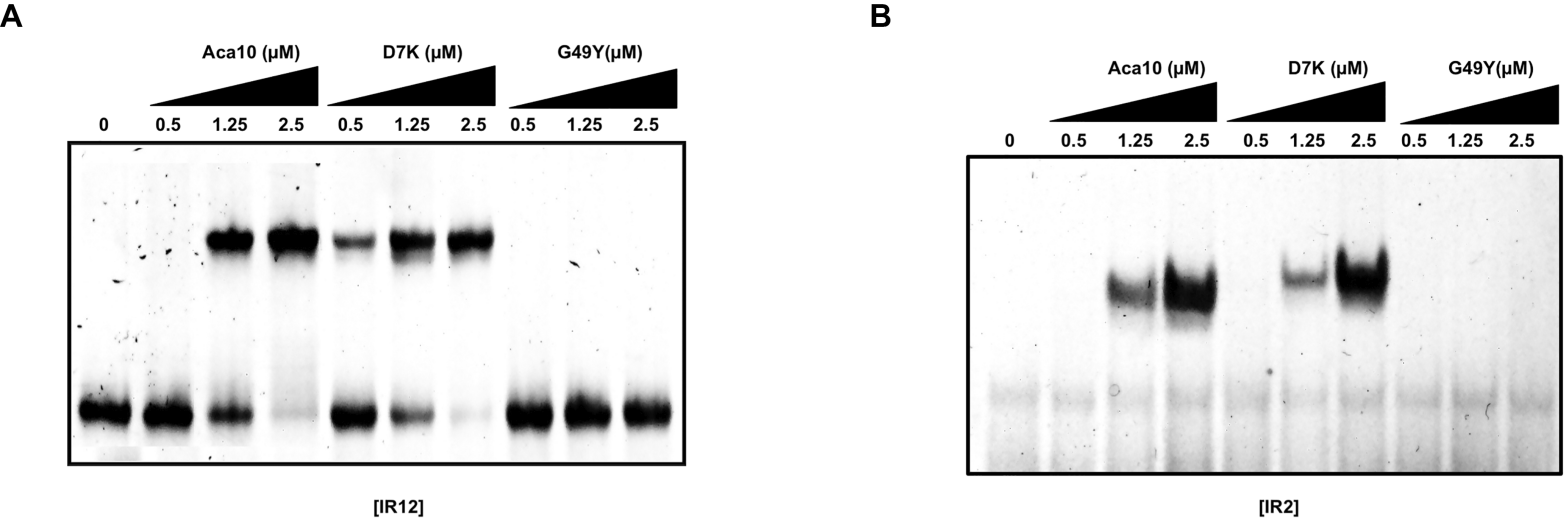
Promoter DNA interaction analysis with dimer disruption mutants. (**A** and **B**) EMSAs of the IR12 DNA probe (A) and IR2 DNA probe (B) with increasing concentrations of dimer disruption mutants of Aca10 indicated by black triangle. DNA probes used in the experiment are indicated below the gel. The concentrations of each mutant as well as wildtype Aca10 are indicated with each lane in the gel.

Inverted repeat binding sites are frequently recognized by dimeric proteins. To determine whether this applies to Aca10 and the dimeric state is essential for target recognition, we performed EMSAs with dimerization-defective mutants of Aca10, G49Y. The G49Y mutant was completely abrogated in its ability to bind to DNA fragments with either both IR1 and IR2 (IR12) or IR2 alone, even at high concentrations (Figure 4A and B). The D7K mutant that has no effect on dimerization was included as a negative control and, as expected, was capable of binding IR12 and IR2 similar to the wild-type Aca10. In conclusion, Aca10 dimerization is critical for the recognition of the specific IR2 DNA sequence in the *acrIC7-acrIC6-aca10* promoter.

### Strategy of promoter recognition by Aca10

**Figure 5.**
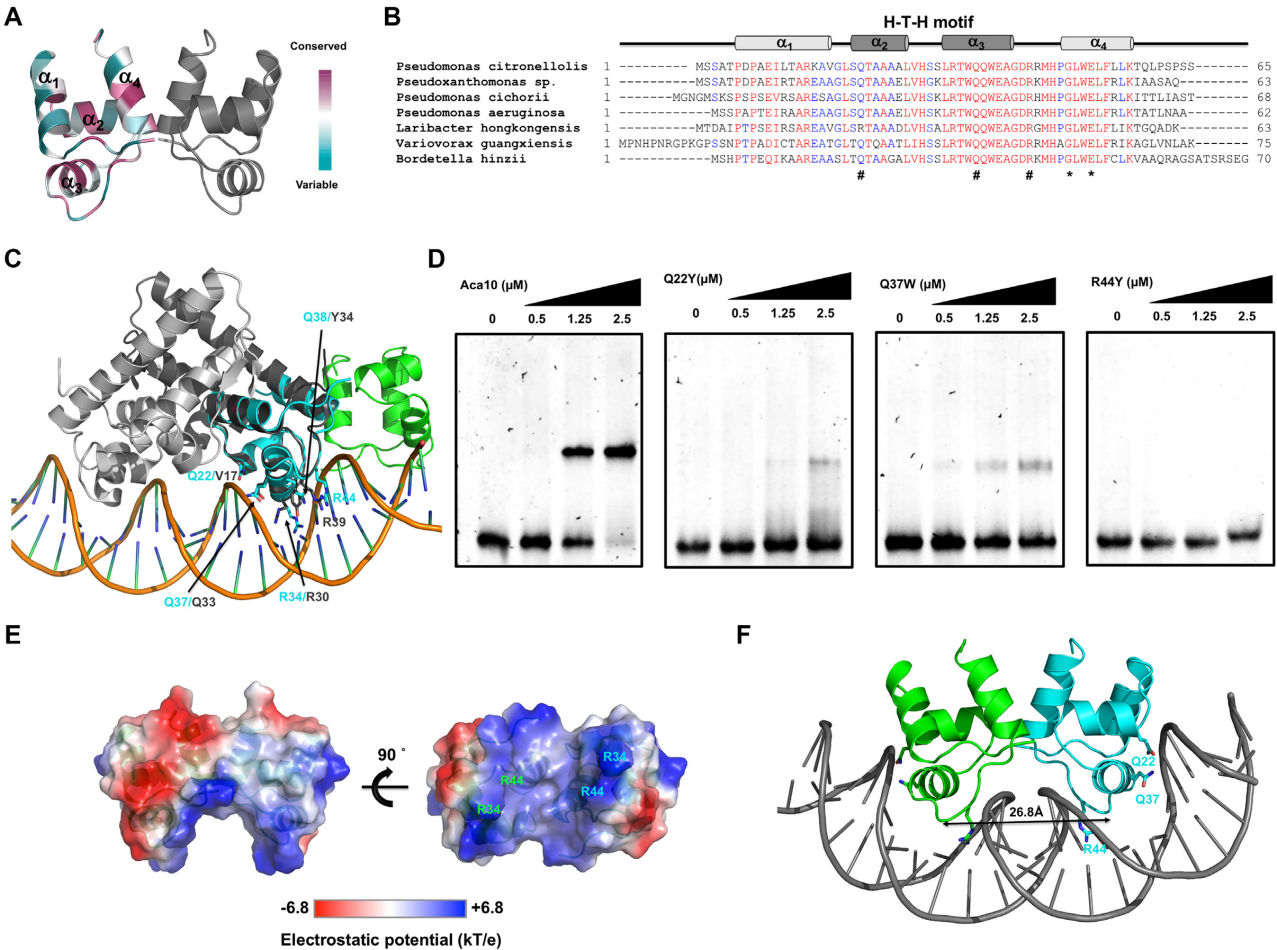
Strategy of promoter recognition by dimeric Aca10. (**A**) Cartoon representation of dimeric Aca10 colored according to the degree of amino-acid sequence conservation (ConSurf). (**B**) Sequence alignment of Aca10 homologs from different species. Mostly conserved and partially conserved residues are colored in red and blue, respectively. The location of the putative H-T-H motif is shown above the sequence. # indicates conserved residues that might be involved in the DNA recognition. * indicates the conserved residues involved in the formation of the dimeric interface. (**C**) Superimposition of the dimeric Aca10 structure with an Aca2/DNA complex structure (PDB id: 7VJQ). Representative residues that might be involved in the DNA recognition were indicated and labeled cyan (Aca10) and gray (corresponding residues on Aca2). (**D**) EMSAs of the IR12 DNA probe with increasing concentration (indicated by black triangles) of putative DNA binding-disturbed mutants, including Q22Y, Q37W, and R44Y. (**E**) Surface electrostatic features of dimeric Aca10. The scale bar ranges from −6.8 kT/e (red) to 6.8 kT/e (blue). (**F**) Structural model of an Aca10/bent DNA complex generated using the HDOCK server. Residues Q22, Q37 and R44, which were experimentally shown to be involved in the readout of the palindromic sequence are labeled in the structure. The distance between two α_3_ helices in the H-T-H motif from both molecules is indicated by a black double headed arrow.

We next investigated how Aca10 recognizes DNA. A combination of sequence- and structure-based comparisons were made to identify residues that might contribute to DNA binding. Analysis of protein conservation (ConSurf) (41) showed that residues in the C-terminus (α_3_, α_4_ and the connecting loop) were more conserved, indicating that this region of Aca10 is likely important for its function (Figure 5A and B). Within the predicted H-T-H motif, the α_2_ residues were less conserved, while α_3_ residues were completely conserved, suggesting that α_3_ is the key helix that interacts with DNA. Interestingly, G49 and E52, which were involved in the dimerization of Aca10, were completely conserved, confirming the importance of dimerization for DNA binding (Figure 5B). To obtain further insights into the molecular basis of Aca10 function, we compared the Aca10 structure with its structural homologs using the DALI server (42). The top five PDB matches were Aca2 (7CQ8), NE0471 (2AUW), SO3848 (2OX6), HigB2 (5JAA) and Aca1 (7C0A) (Table 2). Aca1 and Aca2 are families of anti-CRISPR-associated proteins, whereas NE0471, SO3848 and HigB2 all contained an H-T-H motif for binding nucleic acids.

**Table 2.** Structural similarity search performed using DALI (42)

Proteins (accession numbers)	*Z*-score	RMSD (Å)	Identity (%)	References
Aca2 (7CQ8)	9.7	1.7 (59/120)	24	(28)
NE0471 (2AUW)	9.3	1.4 (57/155)	16	Unpublished
SO3848 (2OX6)	9.2	1.7 (59/163)	15	Unpublished
IGA-2 (5JAA)	9.0	1.9 (58/102)	28	(43)
Aca1 (7C0A)	8.7	1.7 (58/73)	22	(28)

Because the structure of Aca2 in complex with DNA was recently solved (28) and was the most structurally related protein to Aca10 via the DALI search, we superimposed our dimeric Aca10 structure into the Aca2/DNA complex structure. Aca2 is much longer than Aca10 and the sequence identity between the proteins is low (∼24% sequence identity). The Aca10 dimer did not superpose well with the Aca2/DNA complex; however, monomeric Aca10 superposed well with part of one Aca2 monomer (Figure 5C). In particular, the H-T-H motif composed of α_2_−α_3_ of Aca10 perfectly fits the H-T-H motif of Aca2 (Figure 5C). Since residues V17, R30, Q33, Y34 and R39 of Aca2 were identified as important for promoter recognition of Aca2 (18,26,28), we found the corresponding residues on Aca10 by structural and sequence alignment (Q22, R34, Q37, Q38 (Y34 on Aca2), and R44). We examined the role of Q22, Q37 and R44 by generating Q22Y, Q37W and R44Y mutants and assessing their DNA binding ability by EMSA. In agreement with the importance of R39 in Aca2 (28), the R44Y mutant of Aca10 was unable to bind to DNA fragments containing both IR1 and IR2 (Figure 5D) or IR2 alone (Supplementary Figure S1). In contrast, Q22Y and Q37W mutants still bound DNA, but binding was significantly impaired (Figure 5D and Supplementary Figure S1)). Altogether, these experiments indicated that all three residues were important for IR recognition and R44 was the most critical residue for the promoter interaction.

Finally, to examine the promoter binding strategy of Aca10 based on our experiments, we analyzed the electrostatic surface of the putative DNA binding site of the Aca10 dimer and performed structural modeling and docking using the HDOCK server (38). Electrostatic surface analysis revealed a highly positively charged pocket on the underside, formed mainly by R34 and R44, that might correspond to the position of DNA interaction (Figure 5E). Sequence and structural analysis of Aca10 demonstrated that the putative H-T-H motif, a well-known DNA binding motif, was predicted to consist of α_2_, α_3_ and its connecting loop. This H-T-H motif and a long loop that connects α_3_ and α_4_ contribute a major part of this deep bottom groove in the Aca10 dimer. When Aca10 was docked with linear DNA, it was not properly loaded into its target. When Aca10 was docked with 50° bent DNA, it fitted well in the cavity of the bent DNA double helix (Figure 5F). In the top scoring docking model, dimeric Aca10 localized the two major grooves of double strand DNA by inserting α_3_ of the H-T-H motif (Figure 5F) with Q22, Q37 and R44 involved in recognition of the IR sequence. Among the interacting residues, R44 was the major DNA read-out contributor that was localized in the center of the deep major groove (Figure 5F and Supplementary Figure S2), while Q22 was localized in the upper side of the center of the major groove and was marginally involved in the DNA interaction. The distance between each α_3_ of the H-T-H motif from both Aca10 molecules within the dimer was ∼26.8 Å, which was shorter than the case of Aca2, which was approximately 33.8 Å. Because the length of a complete turn of double-helical DNA is ∼34 Å, the distance of 26.8 Å suggests the importance of high DNA bending during recognition by dimeric Aca10.

In conclusion, Aca10 from *P. citronellolis* is a dimeric DNA binding protein and a likely transcriptional repressor of the *acrIC7-acrIC6-aca10* operon. Aca10 forms a dimer in solution and the dimeric form is critical for binding specific DNA. The putative H–T–H motif composed of the α_2_−α_3_ helices of 10 directly recognizes a minimal 19 bp palindromic sequence (5′-ATACGCTCATTGAGCGTAT-3′), which obscures the promoter of the *acr* operon. R44 in the connected loop between α_3_ helix and α_4_ helix is a critical residue involved in the DNA binding, which likely results in a high degree of DNA bending.

## DATA AVAILABILITY

Atomic coordinates and structure factors for the reported crystal structures have been deposited with the Protein Data bank under accession number 7XI5.

## Supplementary Material

gkac656_Supplemental_File
