# Supplementary material for: Molecular basis of anti-CRISPR operon repression by Aca10
Source: Nucleic Acids Res. 2022 Aug 3;50(15):8919–28. doi: 10.1093/nar/gkac656 (PMC9410881; doi:10.1093/nar/gkac656)
Supplement: gkac656_Supplemental_File [file gkac656_supplemental_file.pdf]

Molecular basis of anti-CRISPR operon repression by Aca10

So Yeon Lee[1,2], Nils Birkholz[3,4], Peter C. Fineran[3,4], and Hyun Ho Park[1,2,*]

[1]College of Pharmacy, Chung-Ang University, Seoul 06974, Republic of Korea
[2]Department of Global Innovative Drugs, Graduate School of Chung-Ang University, Seoul 06974, Republic of Korea
[3]Department of Microbiology and Immunology, University of Otago, PO Box 56, Dunedin 9054, New Zealand
[4]Bioprotection Aotearoa, University of Otago, PO Box 56, Dunedin 9054, New Zealand

* To whom correspondence should be addressed. Tel: +82-2-820-5930; Fax: +82-2-820-3033; Email: xrayleox@cau.ac.kr

Supporting Information

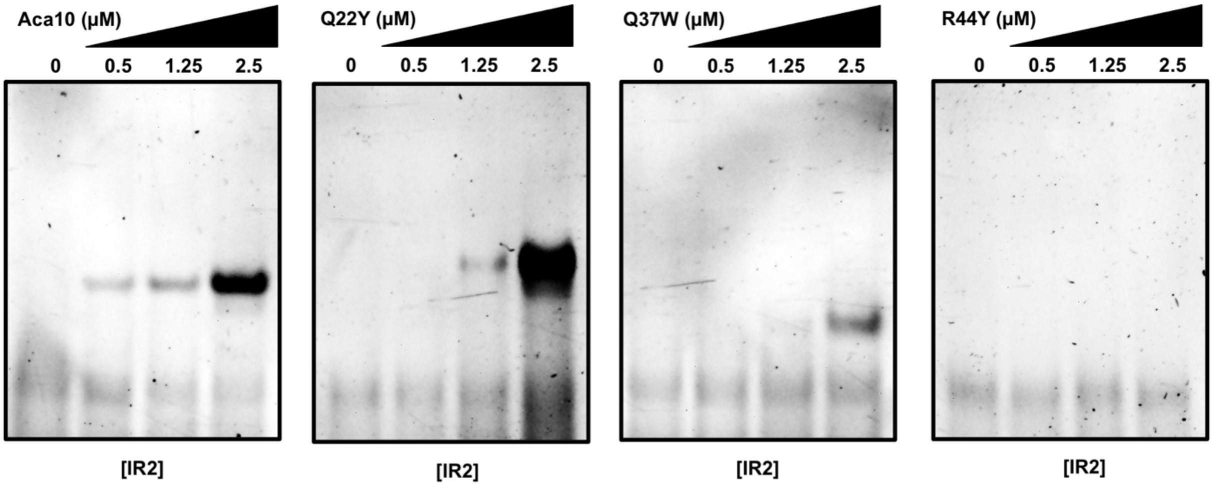

Supplementary Figure 1. EMSAs of IR2 DNA probe with increasing concentration of putative DNA binding-disturbed mutants, including Q22Y, Q37W, and R44Y. DNA probes used in the experiment are indicated below each gel. Concentration-dependency is indicated by the black triangle

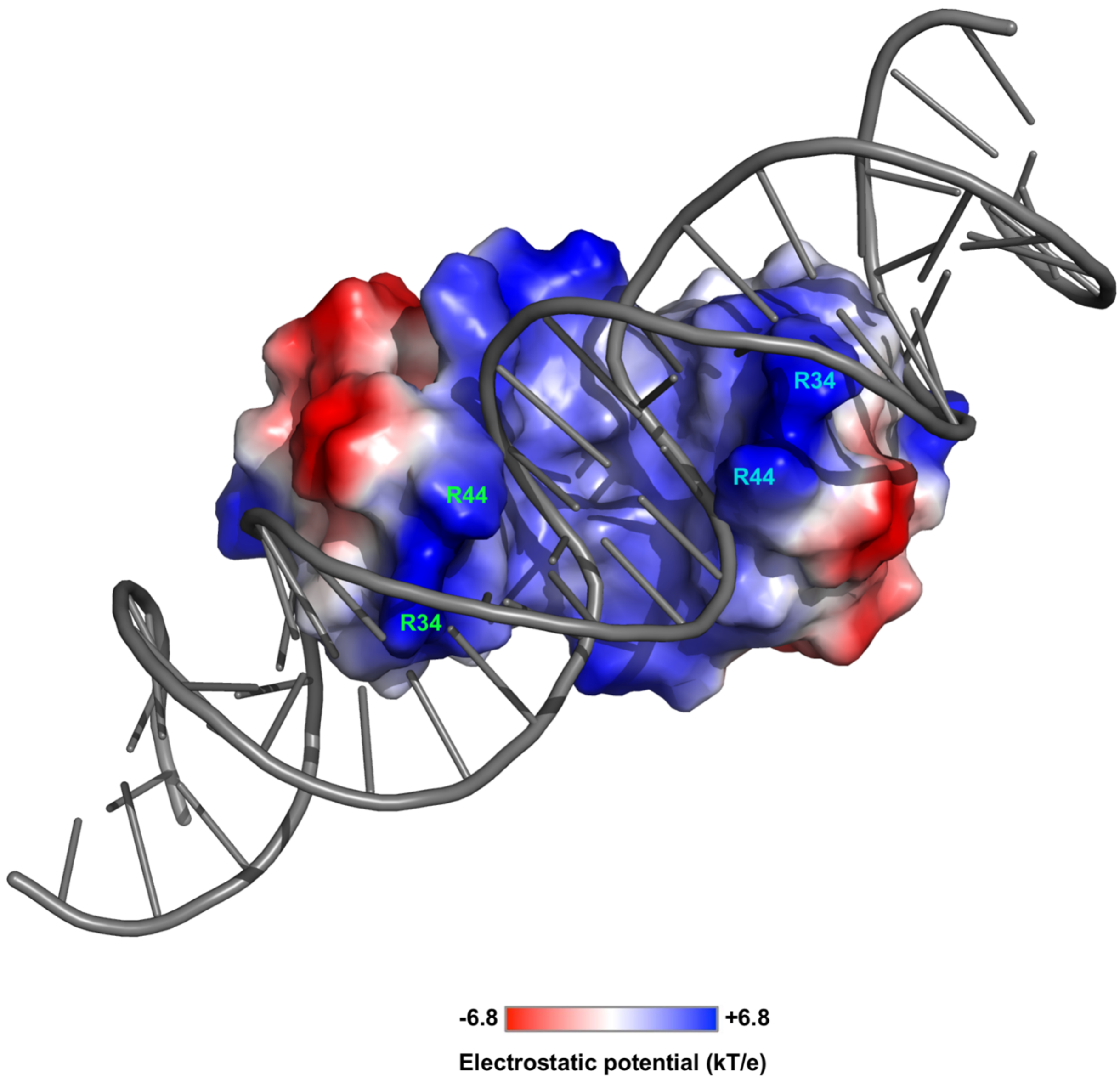

Supplementary Figure 2. Structural model of Aca10/promoter DNA complex view form the bottom of Aca10 dimer. Aca10 was provided by electrostatic surface model. Gray double strands indicate DNA. Critical residues for DNA interaction are labelled.
